# Analysis of Cascading Failure in Gene Networks

**DOI:** 10.3389/fgene.2012.00292

**Published:** 2012-12-14

**Authors:** Longxiao Sun, Shudong Wang, Kaikai Li, Dazhi Meng

**Affiliations:** ^1^College of Information Science and Engineering, Shandong University of Science and TechnologyQingdao, China

**Keywords:** systems biology, gene network, cascading failure, betweenness centrality, structural key gene

## Abstract

It is an important subject to research the functional mechanism of cancer-related genes make in formation and development of cancers. The modern methodology of data analysis plays a very important role for deducing the relationship between cancers and cancer-related genes and analyzing functional mechanism of genome. In this research, we construct mutual information networks using gene expression profiles of glioblast and renal in normal condition and cancer conditions. We investigate the relationship between structure and robustness in gene networks of the two tissues using a cascading failure model based on betweenness centrality. Define some important parameters such as the percentage of failure nodes of the network, the average size-ratio of cascading failure, and the cumulative probability of size-ratio of cascading failure to measure the robustness of the networks. By comparing control group and experiment groups, we find that the networks of experiment groups are more robust than that of control group. The gene that can cause large scale failure is called structural key gene. Some of them have been confirmed to be closely related to the formation and development of glioma and renal cancer respectively. Most of them are predicted to play important roles during the formation of glioma and renal cancer, maybe the oncogenes, suppressor genes, and other cancer candidate genes in the glioma and renal cancer cells. However, these studies provide little information about the detailed roles of identified cancer genes.

## Introduction

As the development of molecular biology and the application of some biological technologies, it has become a hot spot issue in studying different cancers (Hanash, 2004; Rhodes and Chinnaiyan, 2005; Segal et al., 2005) in the view of gene, to reveal the mechanism of formation and development of cancer and look for efficient treatments. The canceration of tissue cells experiences three stages, initiation, development, and diffusion of cancer cells, each of which involves activation of oncogenes and inactivation of suppressor genes. Hence, finding the key genes related with disease characteristics is of great significance to the diagnosis and cure of the cancer and drug design. It is an important project in the research of bioinformatics (Lander and Weinberg, 2000). Now most of the methods researching the immanent mechanism of genome are based on biochemical experiments and only fit for some specific genes. As the fast accumulation of cancer genome data, it becomes possible to make models with a large scale of data. In systems biology, some efficient methods have been explored, such as integrally studying the changing pattern of genome model in the experiment of tumor, through analyzing the interaction network of genes (Alm and Arkin, 2003; Barabasi and Oltvai, 2004) to reveal the biological way of gene function. These methods have widely promoted the study of molecular mechanism in a large extent. For example, as for model organisms yeast, nematode, and fruit fly, computational biologists have used these methods to make a lot of predictions for the function of gene (Jansen et al., 2002; Mateos et al., 2002; Pavlidis et al., 2002; Lee et al., 2004; Zhang et al., 2004; Nabieva et al., 2005; Vidal, 2005; Barutcuoglu et al., 2006).

Cascading failure of complex network is defined as one or a few nodes or links failure which will lead other nodes failure through the coupling relations, and it will cause the chain effect and lots of nodes failure, ever the collapse of the whole network, also vividly called “avalanche.” As human society networking increasingly, people become stricter and stricter with the security and reliability of complex network. People make a lot of effort, but still large scale cascading failures have occurred from time to time. The reliability of complex networks has increasingly become an important issue in internet networks (Cohen et al., 2000; Pastor–Satorras et al., 2001; Goh et al., 2002; Willinger et al., 2002), power grid (Albert et al., 2004; Kinney et al., 2005), and traffic networks (Wu and Sun, 2007). Cohen et al. (2000) studied the internet networks which follow a scale-free power-law distribution with respect to random crashes. Kinney et al. (2005; Cohen et al., 2000) modeled the power grid using its actual topology and plausible assumptions about the load and overload of transmission substations. Wu et al. (2007) studied different removal strategies affect the damage of cascading failures based on the user-equilibrium assignment, which ensures the balance of flp on the traffic network. Smart et al. (2008) investigated the relationship between structure and robustness in the metabolic network of *Escherichia coli*, *Methanosarcina barkeri*, *Staphylococcus aureus*, and *Saccharomyces cerevisiae*, using a cascading failure model based on a topological flux balance criterion.

In this research, we construct mutual information networks using gene expression profiles of glioblast and renal in normal condition and cancer conditions. The method of cascading failure is firstly applied in gene networks to explore the relationship of structure and robustness. The sources of raw gene expression data and the manipulations of the data are presented in Section “Data Source and Processing”. Section “Construct Mutual Information Networks” shows the mutual information gene networks constructed from the processed data sets. Section “Cascading Failure Model” shows the cascading failure model and our main results. A conclusion and discussion section comes to the end of the paper with some open problems.

## Data Source and Processing

### Data source

The sample data of glioma are chosen from GPL570 in NCBI. They are all from GSE4290. The group with cancer called experiment group I, stages II, III, IV include 45, 31, 81 samples respectively. The group without cancer called control group I has 23 samples. The sample data of renal cancer are chosen from GPL570 in NCBI too. The group with cancer called experiment group II, stages I, II, III include 30, 22, 30 samples, and they are all from GSE2109. The group without cancer called control group II has 30 samples which come from GSE11024, GSE12606, GSE3526, GSE7307, and GSE7392 (The detail can be seen in)^1^. Control group I and control group II are called control group. Experiment group I and experiment group II are called experiment group. The detail is shown in Table 1. Each of these data sets includes *p*-values and P-M-A (P, A, and M respectively stand for presence, absence, and margin) for 20,827 genes, corresponding to 54,676 probes.

**Table 1:** **The data source**.

		Data set	Sample size
Control group I		GSE4290	23
Experiment group I	Stage II	GSE4290	45
	Stage III	GSE4290	31
	Stage IV	GSE4290	81
Control group II		GSE11024, GSE12606, GSE3526, GSE7307, GSE7392	30
Experiment group II	Stage I	GSE2109	30
	Stage II	GSE2109	22
	Stage III	GSE2109	30

### Selection of cancer-related genes

Begin with the 54,676 probes above, we first delete probes that corresponding to no gene or more than one gene. There were 20,827 probes left. Next, if more than one probe corresponding to a gene, the expression profile of this gene is determined by the mean value of the profiles of corresponding probes, thus, there were 19,802 genes left. It is too complex to construct and analyze the mutual information networks for all genes in the data sets. So, it is necessary to delete a part of genes and reserve the most important genes. We use Wilcoxon rank sum test to select genes that have obvious differences between control group and experiment groups. Taking glioma for example. Firstly, the control group I and stage II of experiment group I are used with Wilcoxon rank sum test, and we obtain a group of genes charged GI_II. And then the control group I and stage III of experiment group I, the control group I and stage IV of experiment group I is used with Wilcoxon rank sum test, and obtain genes set GI_III and GI_IV respectively. The significance level of Wilcoxon rank sum test is 2.5 × 10^−7^. The intersection of GI_II, GI_III, and GI_IV, that is G1 = GI_II∩GI_III∩GI_IV. The obtained data set G1 is the working data set. We deal with the renal cancer data with the same method in the significance level of wilcoxon rank sum test 1.5 × 10^−8^, and obtain the working data set G2. The rank sum test results are shown in Table 2.

**Table 2:** **The rank sum test results of glioma and renal cancer**.

	Glioma				Renal cancer			
Gene set	GI_II	GI_III	GI_IV	G1	GII_I	GII_II	GII_III	G2
Gene num	182	237	2109	91	953	138	759	106

## Construct Mutual Information Networks

To build a network model for a biological system and make biologically relevant predictions on the function of the system, it is necessary to identify the system’s structure. In this work, we study the structure characteristics of networks consisting of cancer-related genes. A gene expression profile is a vector whose components are its expressions in different experiments. For convenience, we denote gene expression profiles by their corresponding genes. For example, the mutual information of genes *A* and *B* means the mutual information of their expression profiles. The idea of mutual information stems from information theory. It measures dependence degree of two stochastic variables. Let *A* and *B* be two genes (regarded as two stochastic variables). Their mutual information *I*(*A*, *B*) is given by *I*(*A*, *B*) = *H*(*A*) + *H*(*B*) − *H*(*A*, *B*), where $H\left(x\right)=-\sum_{x\in X}p\left(x\right)\log_{2}p\left(x\right)$ is the Shannon entropy of vector *X*. *H*(*X*, *Y*) is the joint entropy of genes *A* and *B*. Larger values of *I*(*A*, *B*) imply closer interrelation between genes’ expressions. In the case of *I*(*A*, *B*) = 0, genes’ expressions are irrelevant.

To calculate relevance of mutual information between genes, we discretize the *p*-values in each data set as follows. (1) Select the range (Min, Max) for *p*-values and divide it into 20 portions such that each portion contains almost the same number of *p*-values. Order the portions in the number order and denote them by 1st, 2nd, …, 20th interval, respectively. (2) Replace the *p*-values in an interval by its labeling value. Obviously, the granularity of our discretization is finer than that of 0–1 discretization. Comparing with the 0–1 discretization, the fine granularity discretization loses less information contained in the original *p*-values. Therefore, it is reasonable to believe that the mutual information networks based on our finer discretization better reflect the nature of the gene regulatory system.

In this article, we construct networks of mutual information using gene expression data in normal tissues and tissues with cancer in every stage. The genes are treated as the nodes and the links between genes as the edges in the networks. The link of two genes can be measured by mutual information value. The greater the mutual information value between two nodes is, the closer the link and the lesser the edge-length is; the lesser the mutual information value is, the more distant the link and the greater the edge-length is. We treated the mutual information network as weighted network (the weighted value is the mutual information value). The distance between two nodes is negatively related to the weighted value. So, we translate the mutual information network into distance network as follow:

$$w_{ij}=\{\begin{array}{cc} \frac{1}{m_{ij}} & \text{if }m_{ij}\neq 0\\ \infty & \text{if }m_{ij}=0 \end{array}$$

Where *m_ij_* is the mutual information of node *i*, *j* in mutual information network, *w_ij_* is the distance of node *i*, *j* in distance network. So, let *G* = (*V*, *E*, *W*) be a complex gene network with node-set *V* = {1, 2, …, *N*}, edge-set *E* and weight-set *W*.

## Cascading Failure Model

### Betweenness centrality

Betweenness was firstly proposed by Freeman in 1979. It is a measure of a node’s centrality in a network equal to the number of shortest paths from all vertices to all others that pass through that node. The betweenness centrality of a node *v* is given by the expression: $g\left(v\right)=\sum_{s\neq v\neq t}\sigma_{st}\left(v\right)/\sigma_{st}$ where σ*_st_* total number of shortest paths from node *s* to node *t* and σ*_st_*(*v*) s the number of those paths that pass through *v*.

Betweenness centrality is a more useful measure of the load placed on the given node in the network as well as the node’s importance to the network than just connectivity. High betweenness centrality scores indicate that a vertex lies on considerable fractions of shortest paths connecting others and plays an important role in the network.

### The cascading failure model

For a given network, suppose that at each time step one unit of the relevant quantity, the information is exchanged between every pair of nodes and transmitted along the shortest path connecting them. The load at a node is then the total number of shortest paths passing through the node (Goh et al., 2001; Newman, 2001; Holme and Kim, 2002). The capacity of a node is the maximum load that the node can handle. The capacity *C_j_* of node *j* is proportional to its initial load *L_j_*, *C_j_* = (1 + α) *L_j_*, *j* = 1, 2, …, *N*, where the constant α ≥ 0 is the tolerance parameter, and *N* is the initial number of nodes. In our research, we define α = 0. When all the nodes are on, the network operates in a free-flow state. But, the removal of nodes in general changes the distribution of shortest paths. The load at a particular node can then change. If it increases and becomes larger than the capacity, the corresponding node fails. Any failure leads to a new redistribution of loads, and, as a result, subsequent failures can occur.

### The algorithm of cascading failure model

Based on the above mentioned definitions and symbols, we present the algorithm of cascading failure model as follows:

(1) Input the weight matrix of complex gene network *G* = (*V*, *E*, *W*).
(2) Calculate initial load $L_{j}^{0}$ of node *j* and its capacity $C_{j}=\left(1+\alpha \right)L_{j}^{0}$, j = 1,2,  …, N, i = 1.
(3) Delete node *i* and its incident edges in the network, *i* = 1, 2, …, *N*.
(4) Calculate the load of every node in the present network and compare the capacity with the load of every node. If the load is lesser than the capacity for every node in the present network, then go to (5), otherwise, delete every node and its incident edges whose load is greater than its capacity, go to (4).
(5) If the size-ratio of cascading failure after deleting node *i* is greater than or equal to the threshold *t_cf_* of network failure, then the network breaks down.
(6) *i* = *i* + 1 If *i* < *N*, then go to (3).

### The judgment of cascading failure

(1) The criteria of a node’s failure.
  (i) If the load of a node is greater than its capacity, then it is called a failure node.
  (ii) If a node becomes an isolated node, then it is called a failure node.
(2) The criteria of a network’s cascading failure.

If the size-ratio of cascading failure ≥*t*_cf_, the network has cascading failure, where *t*_cf_ is the threshold of network failure, and it is a criterion of network failure. In our research, we define *t*_cf_ = 0.5.

### Some important parameters

(1) After deleting node *i*, and causing *s_i_* failure nodes (including node *i*), then *s_i_* is defined as the size of cascading failure of node *i* and *d*_i_ = *s_i_/N* as the size-ratio of cascading failure.
(2) Let $\text{sign}1\left(i\right)=\left\{\begin{array}{cc} 1, & d_{i}\geq t_{cf}\\ 0, & d_{i}<t_{cf} \end{array}\right\}$ then the percentage of failure nodes of the network $p=\sum_{i=1}^{N}\text{sign}1\left(i\right)/N$.
(3) The average size-ratio of cascading failure $R=\sum_{i=1}^{N}d_{i}/N$.
(4) Let $\text{sign}2\left(i\right)=\{\begin{array}{cc} 1, & d_{i}\geq d\\ 0, & d_{i}<d \end{array}$ (*d* is a variable parameter). Then the cumulative probability of size-ratio of cascading failure $P\left(d^{\prime }\geq d\right)=\sum_{i=1}^{N}\text{sign}2\left(i\right)/N$, which indicates the probability of size-ratio *d_i_* of cascading failure greater than *d*.

Obviously, *P*, *R*, and *P*(*d*′ ≥ *d*) are the important parameters measuring the cascading failure scale and the robustness or fragility of network.

**Figure 1:**
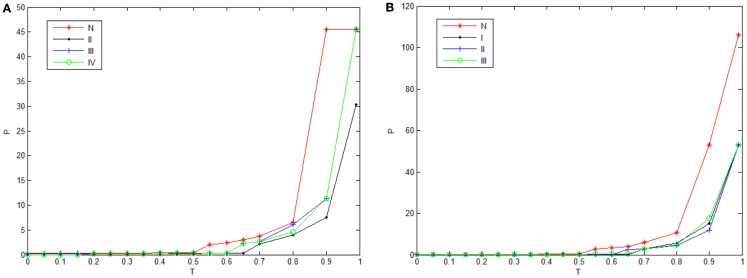
**Plots of the percentage of failure nodes of the network *P* versus threshold values used to construct mutual information networks**. The red, black, blue, and green curves correspond to the cases of the normal state, stage II, stage III, stage IV respectively. **(A)** and **(B)** correspond to glioma and renal cancer.

**Figure 2:**
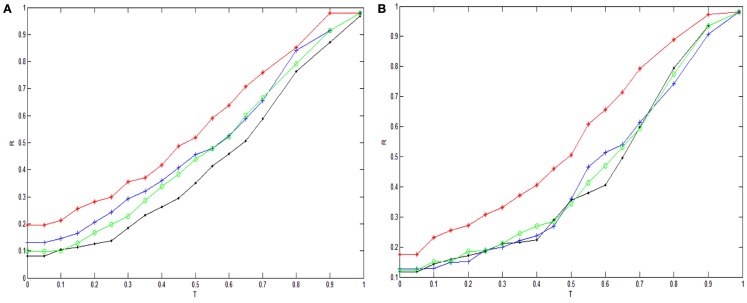
**Plots of the average size-ratio of cascading failure *R* versus threshold values used to construct mutual information networks**. The red, black, blue, and green curves correspond to the cases of the normal state, stage II, stage III, stage IV respectively. **(A)** and **(B)** correspond to glioma and renal cancer.

**Figure 3:**
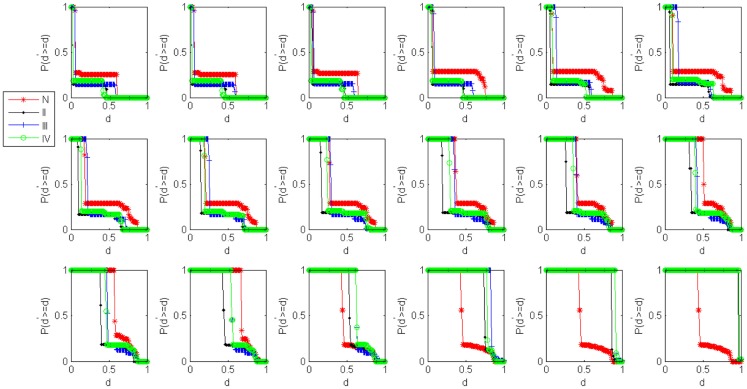
**Damage distributions for cascading events in glioma networks**. Plots of the cumulative probability of size-ratio of cascading failure *P*(*d*′ ≥ *d*) versus the size of cascading failure of node *d* in 18 threshold values. The red, black, blue, and green curves correspond to the cases of the normal state, stage II, stage III, stage IV respectively.

**Figure 4:**
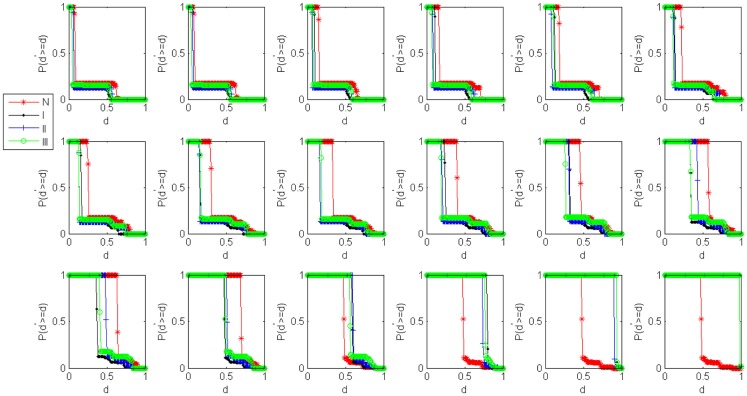
**Damage distributions for cascading events in renal cancer networks**. Plots of the cumulative probability of size-ratio of cascading failure *P*(*d*′ ≥ *d*) versus the size of cascading failure of node *d* in 18 threshold values. The red, black, blue, and green curves correspond to the cases of the normal state, stage I, stage II, stage III respectively.

In order to highlight the structural characteristics of the networks so that valuable biological conclusions can be drawn, it is necessary to choose a threshold value to carry out coarse graining on normalized mutual information. Here, we choose 18 thresholds that are (0, 0.05, 0.1, 0.15, 0.2, 0.25, 0.3, 0.35, 0.4, 0.45, 0.5, 0.55, 0.6, 0.65, 0.7, 0.8, 0.9, 0.99) and then obtain 18 networks correspond to the cases of the normal state and experiment group of every state respectively. The percentage of failure nodes of the network *P* is plotted versus the threshold values used to construct mutual information networks *T* in Figure 1A (glioma) and Figure 1B (renal cancer). The average size-ratio of cascading failure *R* is plotted versus the threshold values used to construct mutual information networks *T* in Figure 2A (glioma) and Figure 2B (renal cancer). In Figures 1 and 2, the control group (red curve) is on the top of every stage of experiment group (black, blue, and green curves) in all values of threshold. The cumulative probability of size-ratio of cascading failure *P*(*d*′ ≥ *d*) is plotted versus the size of cascading failure of node *d* in Figure 3 (glioma) and Figure 4 (renal cancer). In Figure 3, by comparing the networks corresponding to control group I and the stages of experiment group I, one can see that the networks of control group I can be distinguished from the experimental group I clearly in a broad range of the threshold variation that is (0, 0.65). In Figure 4, the networks for control group II can be distinguished from the experimental group II clearly in a broad range of the threshold variation that is (0, 0.55). In addition, the red curve is on the top of the other three color curves. The distinction shows that the differences in the cumulative probability of size-ratio of cascading failure *P*(*d*′ ≥ *d*) for control group and different stages of experiment group are pretty clear. So, we can see from Figures 1–4, the network of control group trends to fail more easily than networks of different disease stages. *P*, *R*, and *P*(*d*′ ≥ *d*) can measure the robustness of the networks, and they are positively correlated with the robustness of the networks.

### The selection of the structural key genes

In the control network and experimental networks, the size of cascading failure of some genes are quite different, and these genes are called structural key genes (SKGs). The situation is very complex, for example gene *AMH* in Table 3, the values of the size of cascading failure are very great in all networks. Gene *MELK* in Table 3, the values of the size of cascading failure are very great in all networks except control group and there are some other genes which have the same characteristic. Tables 3 and 4 list some representative genes which have big difference in the size of cascading failure in different networks of corresponding to glioma and renal cancer (The detail can be seen in)^2^. Tables 5 and 6 list all the type of SKGs and all genes of every type. For example, T_II_III in Table 5 means the genes of this type’s size of cascading failure are very great in networks of state II and state III of experiment group I and little in other networks.

**Table 3:** **List representative genes of glioma which have big difference in the size of cascading failure in different networks**.

State	*T*	*MELK*	*AMH*	*BAT1*	*ID3*
*N*	0.05	0.05	0.60	0.60	0.60
	0.30	0.19	0.88	0.76	0.80
	0.60	0.57	0.88	0.86	0.80
	0.90	0.98	0.98	0.98	0.98
II	0.05	0.47	0.47	0.02	0.02
	0.30	0.57	0.66	0.09	0.10
	0.60	0.57	0.82	0.38	0.40
	0.90	0.87	0.88	0.87	0.87
III	0.05	0.59	0.58	0.05	0.05
	0.30	0.71	0.71	0.21	0.22
	0.60	0.88	0.85	0.47	0.47
	0.90	0.93	0.92	0.91	0.91
IV	0.05	0.42	0.43	0.02	0.45
	0.30	0.67	0.67	0.12	0.45
	0.60	0.87	0.87	0.45	0.45
	0.90	0.92	0.92	0.91	0.91

**Table 4:** **List representative genes of renal cancer which have big difference in the size of cascading failure in different networks**.

State	*T*	*AFM*	*TREH*	*MELK*
*N*	0.05	0.62	0.62	0.08
	0.30	0.62	0.78	0.25
	0.60	0.63	0.84	0.64
	0.90	0.97	0.97	0.97
*I*	0.05	0.50	0.07	0.55
	0.30	0.63	0.16	0.55
	0.60	0.72	0.38	0.55
	0.90	0.93	0.93	0.93
II	0.05	0.07	0.07	0.57
	0.30	0.13	0.13	0.64
	0.60	0.49	0.48	0.64
	0.90	0.91	0.91	0.91
III	0.05	0.55	0.05	0.55
	0.30	0.58	0.13	0.74
	0.60	0.58	0.42	0.84
	0.90	0.93	0.93	0.94

**Table 5:** **List SKGs of glioma**.

Type	Genes
T_II_III	*ADAMTS6*, *KIF4A*, *NDC80*
T_C_II_III_IV	*AMH*
T_II	*ANKFN1*, *CAMK2B*, *EZH2*, *SLC30A3*, *TSPAN11*
T_III_IV	*ANKRD43*, *SST*, *SYNGR3*
T_C	*APOC1*, *BAT1*, *BCL6*, *DPYSL3*, *EIF2C1*, *FCHO1*, *FLJ37464*, *IFI16*, *ILF3*, *IRX3*, *MTHFD2*, *NBN*, *NRXN3*, *PPFIA1*, *PPP1R16B*, *PRRX1*, *SGEF*, *SMARCC1*, *UHRF1*
T_C_IV	*ATP8A2*, *EIF4EBP1*, *ID3*, *MAML2*, *POPDC3*
T_IV	*C14orf94*, *DHRSX*
T_C_III_IV	*C16orf48*
T_III	*CCDC80*
T_II_IV	*HS3ST4*, *KIAA1045*
T_II_III_IV	*CRHBP*, *KIRREL3*, *LGI3*, *MELK*

**Table 6:** **List SKGs of renal cancer**.

Type	Genes
T_C_I_III	*AFM*
T_C	*ALDOB*, *NFKB1*, *SLC12A3*, *SLC22A8*, *SLC22A7*, *TREH*, *RGL3*, *C12orf44*, *SLC13A3*, *C18orf45*, *TTC36*, *LOC283027vLOC727770*
T_I_II_III	*CENPE*, *TYRP1*, *MELK*, *PVRL3*, *C12orf59*
T_C_III	*COL4A1*, *ELF5*
T_III	*CYP17A1*, *MT1H*, *NEK2*, *SERPINA5*, *SPAG4*, *ENPP6vC7orf41*
T_I_III	*DACH1*
T_I	*GPC5*, *TCEAL2*, *LOC100130278*
T_II	*TXNDC3*, *MGC12488*, *GGT6*, *FAM151A*
T_II_III	*MIOX*, *TUBB2B*
T_I_II	*ACSF2*
T_C_I_II	*IYD*

In Table 5, genes in T_II_III, T_II, T_III_IV, T_IV, T_III, T_II_IV, T_III_IV, the size-ratio of cascading failure is great in some experiment group I networks, and very little in control group I. And these genes have great degree and betweenness centrality. That is to say, they are very active in cancer cells but relatively silent in normal cells, and deleting them will cause the collapse of the whole diseased networks. Hence, they are probably glioma oncogenes genes. Genes in T_C are very active in normal cells but relatively silent in cancer cells and, hence, they are probably glioma suppressor genes. Genes in T_C_II_III_IV are the key nodes in both control group I and every states of experiment group I. They are very important during the whole life, not only the normal cells but also the cancer cells. So, we infer these genes are housekeeping genes. Housekeeping genes are constitutively expressed in all tissues to maintain cellular functions. They are presumed to produce the minimally essential transcripts necessary for normal cellular physiology. Genes in T_C_IV, T_C_III_IV, they are the key nodes of control group I, but are not the key nodes in all states of experiment group I, and we have not a clear classification. We can through consulting related data to conform the mechanism in normal and cancer cells. The similar with the Table 5, in Table 6, genes in T_I_II_III. T_III, T_I_III, T_I, T_II, T_II_III, T_I_II are probably renal oncogenes genes. Genes in T_C are probably renal suppressor genes. Genes in T_C_I_III, T_C_III, T_C_I_II need further confirmation.

## Conclusions and Discussions

In these research, we construct mutual information networks using gene expression profiles of glioblast and renal in normal condition and cancer conditions. Translate the mutual information networks into load weighted networks. Investigate the relationship between structure and robustness in the gene networks of the two tissues using a cascading failure model based on betweenness centrality. Calculate the percentage of failure nodes of the network *P*, the average size-ratio of cascading failure *R*, and the cumulative probability of size-ratio of cascading failure *P*(*d*′ ≥ *d*) for the networks corresponding to the control group and experiment groups. As for the percentage of failure nodes of the network *P* and the average size-ratio of cascading failure *R*, the value of *P* and *R* increase with the threshold of the network increasing. On the other hand, they can distinguish the control group network and experiment group networks in all the threshold value. And the value of *P* and *R* of control group network is great than that of experiment group networks. As for the cumulative probability of size-ratio of cascading failure *P*(*d*′ ≥ *d*), the network for control group can be distinguished from the experimental group clearly in a broad range of the threshold variation. And the value of *P*(*d*′ ≥ *d*) of control group network is great than that of experiment group networks. Both the percentage of failure nodes of the network *P*, the average size-ratio of cascading failure *R* and the cumulative probability of size-ratio of cascading failure *P*(*d*′ ≥ *d*) can measure the robustness of the networks, and the value is positively correlated with the robustness of the networks. In terms of structure, the network of control group trends to fail more easily than networks of different disease stages. So we infer the networks of different disease stages are more robust than that of control group to some extent. Kitano (2004) presented a perspective on cancer as a robust system to provide a framework from which the complexity of tumors can be approached to yield novel therapies. The reason why many approaches to anticancer treatment had been limited success was because the tumor was “robustness.” With the growth of threshold, there are some isolated nodes, and links among are no so connected. It is obvious that the scale of cascading failure is more and more great. And the networks are not so robust.

According to the differences of the size of cascading failure of some genes in the control network and experimental networks, we get some SKGs. And we group them into different types. In Table 6, we infer genes in T_C are suppressor genes of glioma, genes in T_II_III, T_II, T_III_IV, T_IV, T_III, and T_II_IV are oncogenes of glioma. Overexpression of the polycomb group protein enhancer of zeste homolog 2 (*EZH2*) occurs in diverse malignancies, including prostate cancer, breast cancer, and glioma (Bachmann et al., 2006; Yu et al., 2007; Simon and Lange, 2008). It is believed to play a crucial role in tissue-specific stem cell maintenance and tumor development. *EZH2* is strongly expressed in glioma samples and its pharmacologic inhibition impairs glioma cells self-renewal *in vitro* and delays tumor initiation *in vivo* (Suvà et al., 2009). In Table 6, most of the genes have not been proved to have direct relationship with glioma, but some of them have significant relationship with other cancers. Riemann et al. (2006) research the association of the *NFKB1* insertion/deletion promoter polymorphism with survival in colorectal and renal cell carcinoma as well as disease progression in B-cell chronic lymphocytic leukemia, and proved that the *NFKB1* promoter polymorphism has no effect on risk and course of disease in the three cancer entities that were analyzed. Okamoto et al. (2011) identified *GPC5* as a new susceptibility gene for nephrotic syndrome and implicated *GPC5* as a promising therapeutic target for reducing podocyte vulnerability in glomerular disease. This research provides a large amount of SKGs, which are key roles in normal tissues and cancer tissues of glioblast and renal. However, this study provides little information about the detailed roles of identified cancer genes. Most of the genes have not been studied the relationship with glioma and renal cancer. The results can predict more detailed and interpretable roles of oncogenes and other cancer candidate genes in glioma and renal cancer.

## Conflict of Interest Statement

The authors declare that the research was conducted in the absence of any commercial or financial relationships that could be construed as a potential conflict of interest.

## Footnotes

^1^http://cise.sdust.edu.cn/institute/isbbc/data/cascading/dataset1.rar

^2^http://cise.sdust.edu.cn/institute/isbbc/data/cascading/dataset2.rar
